# Neglecting the neglected: Tobacco cessation support is essential for the management of asthma and COPD

**DOI:** 10.18332/tid/176228

**Published:** 2024-01-23

**Authors:** Dilek Karadoğan, İlknur Kaya, Merve Yumrukuz Şenel, Esin Bilgin Konyalıhatipoğlu, Tahsin Gökhan Telatar, Metin Akgün

**Affiliations:** 1 Department of Chest Diseases, School of Medicine, Recep Tayyip Erdoğan University, Rize, Türkiye; 2 Department of Chest Diseases, School of Medicine, Kütahya Health Sciences University, Kütahya, Türkiye; 3 Department of Chest Diseases, School of Medicine, Balıkesir University, Balıkesir, Türkiye; 4 Department of Public Health, School of Medicine, Recep Tayyip Erdoğan University, Rize, Türkiye; 5 Department of Chest Diseases, School of Medicine, Ağrı İbrahim Çeçen University, Ağrı, Türkiye

**Keywords:** asthma, COPD, management, smoking cessation

## Abstract

**INTRODUCTION:**

Asthma and COPD management have a broad framework, and smoking cessation plays an essential role. We examine the management of asthma and COPD patients not only for inhaler treatment options but also for essential interventions, such as smoking cessation support.

**METHODS:**

Data were collected cross-sectionally from pulmonology departments of three government hospitals in Türkiye between May and September 2022. Patients aged ≥18 years who had been diagnosed with asthma or COPD for at least a year, were included in the study. The demographic and clinical characteristics of the patients were investigated. Routine cessation interventions were implemented for current smokers, and they were followed via phone calls after one month regarding their quit status and access to cessation clinics.

**RESULTS:**

Data from 145 patients with asthma and 148 patients with COPD were analyzed. The rate of current smoking among patients with asthma and COPD was 18.8% and 34.5%, respectively. Current smoking was negatively associated with age (<65 years) and disease duration (years) for both diseases (p<0.05). In addition, for asthmatics, presence of pulmonary disease in the family (OR:0.28, 95% CI: 0.10-0.79) and for COPD patients presence of hospitalization (OR: 0.26, 95% CI: 0.07-0.93) were negatively associated with current smoking. After one month, 85.1% of current asthmatic smokers had not tried to call a quitline, while 14.8% had tried to contact a quitline. Among current smoker COPD patients, only 1.9% had visited a smoking cessation clinic.

**CONCLUSIONS:**

Tobacco cessation support seems to be neglected in asthma and COPD management. Instead, pulmonologists and patients focus on pharmaceutical treatments, which constitute the other component of care.

## INTRODUCTION

Asthma and chronic obstructive pulmonary disease (COPD) are the most common airway diseases^1^. For their management, inhaler treatment options have a great role in helping to relieve symptoms and improve quality of life. Additionally, avoidance of risk factors that play a role in the etiopathogenesis and triggering of airway diseases, is essential for their management^2^. Among the risk factors, tobacco smoke exposure is a major problem that complicates the management of airway diseases. Furthermore, avoidance of tobacco smoke exposure has been shown to be an unquestionable factor in extending the survival of patients with mild and moderate COPD^3^. Despite this, studies show that approximately 50% of patients with a COPD diagnosis continue to smoke^4,5^, and for asthmatics, that rate was found to be about 20% and 11.4%, in international and national studies, respectively^6,7^. In addition, current smoker patients were found to be more symptomatic and more likely to overuse inhaler treatments^8,9^.

Tobacco cessation is considered to be an essential intervention for these patients^10,11^. It is known that the rate of quitting smoking with the help of smoking cessation support is higher than with unaided attempts^12^. The rates of quitting smoking with an intensive smoking cessation program in asthma and COPD patients, were found to be similar to the control group^13^. The lack of access to evidence-based cessation methods is still cited as a barrier to smokers’ attempts to quit, as 70% of smokers want to quit, while only one-third have used these methods^14^. In this context, smoking cessation clinics offer evidence-based support and the availability of free cessation medications encourages smokers to visit these clinics^15^. However, the availability of and access to smoking cessation clinics for asthma and COPD patients is an unmet need that warrants research.

In light of the gaps in the existing literature, the primary aim of this study was to investigate the current characteristics, smoking habits, and treatment strategies of individuals with chronic airway diseases in real-world settings in order to provide relevant information for the clinical management of these patients. The secondary aim was to explore the feasibility and limitations of routinely implementing smoking cessation support for asthma and COPD patients who are current smokers.

## METHODS

### Study design

This multicenter, descriptive, and questionnaire-based follow-up study was conducted between May and September 2022. Ethical approval was obtained from the Institutional Review Board of the Recep Tayyip Erdoğan University.

### Sample size calculation

Sample sizes were calculated separately for asthmatics and patients with COPD. The two main parameters that were compared for each group were the status of attempting to apply to the smoking cessation outpatient clinic (tried or not) and smoking status (current, never and former smoker). Power analysis was performed using G Power software, and it was calculated that a minimum of 145 patients should be reached for each group with an effect size of 0.3, an alpha error value of 0.05, and a power of 95% with a degree of freedom of 1.

### Inclusion and exclusion criteria

Patients who have had a COPD or asthma diagnosis for at least a year, patients who applied to pulmonology outpatient clinics, and patients aged ≥18 years, were included in the study. Patients with impaired cognitive functions and patients who refused to participate in the study were excluded.

### Data collection

Data were collected from three health institutions, two tertiary and one secondary care. At the admissions, a questionnaire was used to collect data. The questionnaire consisted of five main parts. The first part included demographic characteristics (age, gender, education level, income level, marital status), the second part was about past medical history (comorbidities, age at diagnosis for airway disease, inhaler/or other medication use), and the third part was for recording the laboratory and function tests. The following part focused on smoking habits and the type of tobacco product used, which was a combustible cigarette for our sample. Current smokers receive counselling that is based on the 5As brief cessation interventions: Ask about smoking habits; Advise patient to quit smoking; Assess offering assistance; Assist with a quit plan; and Arrange by scheduling follow-up. All current smokers were directed to make an appointment at smoking cessation clinics, which offer counselling as well as free cessation medications. As a result, the final section of the questionnaire was a brief follow-up (one month later) with data on current smokers’ compliance with quitting advice.

### The scales used

The modified Medical Research Council dyspnea scale (mMRC) was used and scored from 0–4, and the GOLD classification was performed for COPD according to the final guideline. According to the novel COPD guideline (GOLD 2023), patients were categorized depending on the classification scheme A to E. Category A represents the milder symptom burden, and E represents the severest. The COPD assessment test (CAT) score was calculated for COPD patients, and the asthma control test (ACT) and asthma severity were calculated for asthma patients^10,11^. The smoking status was classified as ‘never’, ‘former’ and ‘current’.

### Statistical analysis

Processing and statistical evaluations of all the data in our study were carried out using the IBM SPSS Statistics 25.0 (SPSS, Inc., Chicago, IL, USA) package program. Differences between the groups were determined by Student’s t-test or Mann Whitney U test for numeric variables, and the relations between categorical variables were determined by chi-squared analysis. A p<0.05 was considered as significant. All tests were two-tailed. Factors affecting current smoking were determined by multivariable logistic regression without adjustment. All independent variables in Table 1 were analyzed by airway disease, and statistically significant covariates, as well as asthma control level and COPD disease categories, were included in the regression models. Results are presented as odds ratios (ORs) and 95% CI.

**Table 1:** Comparison of demographic characteristics of asthma and COPD patients, multicenter cross-sectional study, May–September 2022, Türkiye (N=293)

*Characteristics*	*Asthma (N=145) n (%)*	*COPD (N=148) n (%)*	*p*
**Age**, median (IQR)	47.50 (9.25)	63.00 (7.75)	<0.001
**Age** (years)			<0.001
<65	109 (75.2)	66 (44.6)	
≥65	36 (24.8)	82 (55.4)	
**BMI**, median (IQR)	29.38 (8.38)	25.71 (6.85)	<0.001
<30	80 (55.2)	120 (81.1)	<0.001
≥30	65 (44.8)	28 (18.9)	
**Gender**			<0.001
Female	115 (79.3)	10 (6.8)	
Male	30 (20.7)	138 (93.2)	
**Marital status**			0.024
Married	113 (77.9)	130 (87.8)	
Single	32 (22.1)	18 (12.2)	
**Job**			<0.001
Housewife	97 (66.9)	9 (6.1)	
Retired	8 (5.5)	72 (48.6)	
Working actively	40 (27.6)	72 (48.6)	
**Education level**			0.028
Primary school and lower	111 (76.6)	128 (86.5)	
At least high school graduate	34 (23.4)	20 (13.5)	
**Income level**			0.034
Low (≤ minimum wage)	26 (17.9)	42 (28.4)	
Middle-high (> minimum wage)	119 (82.1)	106 (71.6)	
**Smoking status**			<0.001
Never smoker	96 (66.7)	11 (7.4)	
Former smoker	21 (14.6)	86 (58.1)	
Current smoker	27 (18.8)	51 (34.5)	
**Pack-years** (N=185), median (IQR)	20.00 (18.50)	50.00 (21.25)	<0.001
**Fagerström score**, median (IQR)	5.00 (4.00)	5.00 (4.00)	0.168
Previous quit attempts*	17 (62.9)	26 (50.9)	0.569
**Smoking cessation clinic admission in previous quit attempts**	8 (29.6)	11 (21.5)	0.053
**Smoking initiation age**, median (IQR)	19.00 (7.75)	16.00 (5.00)	0.076
**Presence of comorbidities**			
Metabolic diseases	62 (42.7)	41 (27.7)	0.285
Malignancy	2 (1.4)	7 (4.7)	0.019
Cardiovascular diseases	58 (40.0)	61 (41.2)	0.017
Bronchiectasis	1 (68.0)	15 (10.1)	<0.001

* Out of 27 asthmatics and 51 COPD patients. BMI: body mass index (kg/m^2^) . IQR: interquartile range.

## RESULTS

### Demographic characteristics

Those with asthma-COPD overlap syndrome (3 patients) and pure bronchiectasis (8 patients) were excluded from a total of 304 patients with chronic airway disease. Data from 145 asthma and 148 COPD patients were evaluated. Table 1 summarizes the comparison of their characteristics. The median age of COPD patients was higher than that of asthmatics (63.0 vs 47.5 years), and 55.4% of COPD patients and 24.8% of asthmatics were aged ≥65 years. Asthmatics had a higher median body mass index (BMI, kg/m^2^), and 44.5% of asthmatics and 18.9% of COPD patients were obese. COPD patients were predominantly male (93.2%), while asthmatics were predominantly female (79.3%). Most asthmatics were housewives (66.9%), whereas the majority of COPD patients were either actively employed (48.6%) or retired (48.6%). Asthma patients were predominantly non-smokers (66.7%), whereas COPD patients were ex-smokers (58.2%). The rate of current smoking was 18.8% among asthmatics and 34.5% among COPD patients. Asthmatics have a lower median pack-years than COPD patients (p<0.05). Higher rates of cancer and cardiovascular disease (CVD) were associated with COPD. COPD was diagnosed at an older median age.

### Comparison of current smokers

Factors associated with current smoking in multivariate analysis in asthma and COPD patients are presented in Table 2. Among asthma patients, younger age (<65 years) was found to be positively associated with current smoking compared to the elderly (OR=12.18; 95% CI: 1.47–100.70). Pulmonary disease in the family (OR=0.28; 95% CI: 0.10–0.79) and each unit increase in the diagnosis time (OR=0.90; 95% CI: 0.83–0.98) were negatively associated with current smoking for asthma patients. In COPD patients, being younger than 65 years was positively associated with current smoking (OR=3.58; 95% CI: 1.69–7.58) compared to being older. At least one hospitalization in the last year (OR=0.26; 95% CI: 0.07–0.93), and each unit increase in diagnosis duration (OR=0.92; 95% CI: 0.86–0.98), were negatively associated with current smoking.

**Table 2:** Associated variables with current smoking of asthma and COPD patients

*Asthma*	*Univariate analysis*			*Multivariate analysis*	
	*Current smoker (N=27) n (%)*	*Former/never smoker (N=118) n (%)*	*p*	*OR (95% CI)*	*p*
**Age** (years)			0.005		0.020
<65	26 (96.3)	83 (70.3)		12.18 (1.47–100.70)	
≥65	1 (3.70)	35 (29.9)		Ref	
**Comorbid diseases**			0.026		0.280
Present	14 (51.9)	87 (73.7)		0.57 (0.21–1.57)	
Absent	13 (48.1)	31 (26.3)		Ref	
**Family history of any pulmonary diseases**			0.023		0.016
Present	8 (29.6)	63 (53.8)		0.28 (0.10–0.79)	
Absent	19 (70.4)	54 (46.2)		Ref	
**Asthma control**			0.486		
Very poorly controlled	9 (33.3)	32 (27.1)		Ref	
Not-well controlled	4 (14.8)	30 (25.4)		1.57 (0.58–4.27)	0.373
Well controlled	14 (51.9)	56 (47.5)		0.18 (0.02–1.68)	0.135
**Diagnosis duration** (years), median (IQR)	3 (9)	9.5 (11)	0.006	0.90 (0.83–0.98)	0.024
***COPD***	***Univariate analysis***			***Multivariate analysis***	
	***Current smoker (N=51) n (%)***	***Former/never smoker (N=97) n (%)***	***p***	***OR (95% CI)***	***p***
**Age** (years)			<0.001		0.001
<65	33 (64.7)	33 (34.0)		3.58 (1.69–7.58)	
≥65	18 (35.2)	64 (65.9)		Ref	
**Exacerbations requiring hospitalization**			0.006		0.038
0	47 (92.2)	71 (73.2)		Ref	
≥1	4 (7.8)	26 (26.8)		0.26 (0.07–0.93)	
**GOLD**			0.608		0.595
A	20 (39.2)	34 (35.0)		Ref	
B	10 (19.6)	15 (15.4)		1.32 (0.46–3.74)	
E	21 (41.1)	48 (49.4)		1.41 (0.57–3.48)	
**Diagnosis duration** (years), median (IQR)	4 (6)	8 (8)	0.001	0.92 (0.86–0.98)	0.023

Factors affecting current smoking were determined by multivariable logistic regression without adjustment. IQR: interquartile range.

### Outcomes of brief smoking cessation intervention

Among current smokers, 23 (85.1%) of 27 current asthmatic smokers did not attempt to call any quitline, while 4 (14.8%) did attempt to contact a quitline in the following month. During that one-month time, among 51 current smokers with COPD, 31 (60.7%) did not attempt to call quitlines, 19 (37.2%) did attempt to contact quitlines, and just 1 (1.9%) was admitted to a smoking cessation clinic.

## DISCUSSION

According to our data, 18.8% of asthmatics and 34.5% of COPD patients were current smokers in real-world settings. Over 95% of the sample, including smoker and non-smoker patients, were experiencing the burden of current airway disease and using at least one inhaler device for symptom relief. Current smoking was negatively associated with age (<65 years) and disease duration for both diseases. In addition, for asthmatics, presence of pulmonary disease in the family and for COPD patients presence of hospitalization were negatively associated with current smoking. All smokers were advised to apply SCCs as part of usual care; however, only one of them had achieved it in the following month, and the rest were all continuing to smoke.

In this study, the demographic and clinical characteristics of asthma and COPD patients admitted to real-life settings were also analyzed in detail. Differences were observed in many aspects, the most prominent being gender and tobacco exposure, as expected. In global studies, there is a male predominance for COPD and a female predominance for asthma. However, the difference is not as obvious as in our study. The role of gender roles and varying exposures are important in the etiopathogenesis of these diseases^16,17^. For example, 66% of asthmatics are never smokers in our sample, and if we take into account that 79.3% of asthmatics are women, other environmental exposures such as domestics come to the fore^18^. On the other hand, 93.2% of COPD patients were men, and the prevalence of smoking in Türkiye is more than twice that of women^19^.

Another point of view is the related factors for current smoking of both airway disorders; younger age was connected with current smoking in both asthma and COPD, when compared to the elderly (aged ≥65 years). Current smoking was negatively related to having at least one hospitalization in the previous year for COPD. Current smoking is also inversely associated with an increase in the duration of the first diagnosis each year. All of these findings are consistent with prior similar investigations^4^. They continue to smoke throughout the early phases of the disease when the clinical manifestations are still moderate. This may also be related to a poor perception of disease^20^. In addition to commencing symptom-relieving medication for symptomatic patients during these periods, comprehensive education about the disease, the potential triggers, risk factors and smoking cessation support should be initiated.

Support for smoking cessation should be considered as a first step in current smoker asthma and COPD patients. These patients are routinely offered a brief cessation intervention and advised that they can arrange a smoking cessation appointment through the quitlines. However, according to our remote follow-up after one month, just one of the current smoker patients had applied to the smoking cessation outpatient clinic; the remainder were unable to contact the quitlines or did not make any effort to reach the quitlines, and so continued smoking. At that moment, novel cessation approaches can be a solution for this vulnerable group: immediate cessation support, regardless of smoker patients’ preparedness^21,22^. Effective counselling, by following the cessation medication as well as following with or without remote follow-up, was shown to be more effective than the routine approach^23^. The purpose of ‘teachable moments’, such as the diagnosis of a tobacco-related illness, is to encourage smokers to stop; consequently, healthcare practitioners of patients with chronic respiratory disorders should use these opportunities effectively.

In this study, 18% of asthmatics and 34% of COPD patients were current smokers and using their inhaler treatment. Cigarette smoking was found to be associated with inhaler overuse^9^, as well as inhaler non-adherence^8,24^. It is also possible that symptom burdens are greater in current smokers, so bronchodilator inhalers are used to alleviate symptoms such as dyspnea. In a recent trial, COPD patients were given a triple combination of bronchodilators in a single device, and those who quit smoking were compared to those who continued to smoke. Despite the triple inhaler, individuals who continued to smoke experienced no improvement in their symptoms or functional status, and their problems persisted^25^. These findings might be interpreted as follows: continuing to smoke diminishes the efficacy of inhaler therapy, and the optimal strategy is to initiate an effective smoking cessation treatment simultaneously with inhaler therapy. Also, symptomatic tobacco-exposed patients with preserved ratio impaired spirometry do not benefit from dual bronchodilators and smoking cessation therapy is recommended as the primary target for this patient population^26^. Also, for asthmatics, even the presence and amount of former smoking history has been associated with airway eosinophilic inflammation and lower response rates to systemic corticosteroids^27^. Providers of healthcare should thoroughly explain to smokers with chronic airway disorders that the therapy will not be effective unless they quit smoking, and this should be reiterated anytime teachable occasions present themselves.

There are approximately 300 smoking cessation services in the country that offer free counselling and medication^28^. However, in a novel study, it was reported that 21% of smokers are unaware of the presence of smoking cessation clinics, and half did not know what services are provided. In addition, participants reported access barriers to smoking cessation clinics, such as distance to the neighborhood and transportation costs^29^. Similarly, according to our findings, among current smokers, four patients with asthma and nineteen patients with COPD attempted to call the quit line, but only one patient applied to a smoking cessation clinic in the first month. There is a lack of data on the effectiveness of immediate cessation support for this group of individuals with chronic respiratory illnesses, and there is a need for well-designed studies on this group of patients.

### Strengths and limitations

This study is an important reference for assessing real-life situations and identifying problems, and detailed data were collected by pulmonologists. In addition to examining and comparing two of the most common chronic airway diseases in terms of clinical features, therapeutic options, smoking status, and follow-up data within their current smoker subgroup, our findings are informative. Important findings are also revealed by remote follow-up data one month following standard brief cessation therapies for smokers. The fact that symptomatic patients continued to smoke and only one patient applied to smoking cessation outpatient clinics demonstrates that the issue of smoking cessation continues to be undervalued in clinical practice. Due to the cross-sectional design used for the study, causal inferences cannot be made, and the specific site data limit generalizability to other countries. Other limitations of our study are that we did not assess patients’ adherence to inhaled medications and inhaler use, and we did not assess other putative triggers controlling the disease.

## CONCLUSIONS

In the care of asthma and COPD patients in the real world, there exist inadequate practices and neglected circumstances. Our data may be helpful for conducting due diligence and identifying issues so that remedies can be found. Combining quantitative and qualitative methodologies to study the underlying causes of treatment adherence is necessary. In addition, novel tobacco cessation recommendations must be developed to provide current smokers with more effective cessation support.

## Data Availability

The data supporting this research are available from the authors on reasonable request.
