# Supplementary material for: Neglecting the neglected: Tobacco cessation support is essential for the management of asthma and COPD
Source: Tob Induc Dis. 2024 Jan 23;22:10.18332/tid/176228. doi: 10.18332/tid/176228 (PMC10804862; doi:10.18332/tid/176228)
Supplement: Supplementary file 1 [file TID-22-22-s1.pdf]

Inhaler use of the sample

Asthmatics mostly used a single inhaler device (57.7%), whereas COPD patients used multiple devices (62.6%). The use of ICS-containing regimens was 95.2% in asthmatics and 61.5% in COPD patients. Only metered-dose inhaler (MDI) users had a higher rate of asthma (16.9% vs 4.2%), and only dry powder inhaler (DPI) users had a higher rate of COPD (61.5%). Asthmatics were mostly using an inhaled corticosteroids and long-acting beta-2 agonist combination (ICS+LABA) regimen (88.3%), while COPD patients were mostly using triple combinations (43.2%).

Disease severity and stage of the patients

Asthmatics were mostly moderate (55.9%), with mild and difficult-to-treat asthmatics accounting for 25.5% and 18.6%, respectively. According to the ACT, 28.3% of asthma cases were poorly controlled, 23.4% were not- well controlled, and 48.3% were well controlled.
In terms of COPD, 35.8% were in Category A, 29.7% were in Category B, and 34.4% were in Category E. Inhaler bronchodilator treatment regimens for COPD patients classified as GOLD is seen in Supplement table. LABA plus long-acting muscarinic antagonist (LAMA) regimes were most commonly used (43.1%) in category A patients, followed by triple combination with ICS (LABA+LAMA+ICS) (27.5%) and LABA plus ICS (19.6%). Most people in category B were on triple therapy (54%) or LABA plus ICS (18.1%). Nearly half of those in Category E were on triple therapy (LABA+LAMA+ICS) (49%) or LABA plus LAMA (11.3%) or LABA plus ICS (11.3%) (Supp Figure).

Captures of figures:

Figure 1. Distribution of COPD patients according to treatment choices and the categories
Abbrevations: SABD: Short-acting bronchodilator, LABA: Long acting beta-2 agonist, LAMA: Long-acting muscarinic antagonist, ICS: Inhaled corticosteroid

Figure 2. Among current smokers number of patients regarding the use of quit services at first month follow-ups by phone call.

Supplement table. Comparison of clinical and medication use characteristics of asthma and COPD patients.

	Asthma (n:145)	COPD (n:148)	p
**Age at diagnosis, median (IQR)**	44.6 (14.4)	56.8 (10.2)	<0.001
**Diagnosis duration, median (IQR)**	7.00(11.5)	5.00 (8.0)	0.052
**İnhaler users**	142 (97.9%)	142 (95.9%)	0.325
**İnhaler device number**			0.001
**Single inhaler users**	82 (57.7%)	54 (37.8%)	
**Multiple inhaler users**	60 (42.3%)	89 (62.2%)	
**İnhaler device number, (median, IQR)**	2.00 (1.00)	2.00 (1.00)	<0.001
**ICS users**			<0.001
**Present**	138 (95.2%)	91 (61.5%)	
**Absent**	7 (4.8%)	57 (38.5%)	
**Only short acting users**			0.541
**Yes**	4 (2.8%)	6 (4.1%)	
**No**	141 (97.2%)	142 (95.9%)	
**Inhaler types**			0.003
**All/Only nebuliser users**	2 (1.3%)	3 (2.0%)	
**Only Dry powder users**	67 (47.2%)	88 (61.5%)	
**Only MDI users**	24 (16.9%)	6 (4.2%)	
**DPI+MDI users**	49 (34.5%)	46 (32.2%)	
**Montelucast users**	78 (53.8%)	6 (4.1%)	<0.001
**Theophyline users**	2 (1.4%)	7 (4.7%)	0.097
**Long acting group**			<0.001
**None**	7 (4.8%)	10 (6.8%)	
**ICS only**	2 (1.4%)	3(2.0%)	
**LAMA only**	0	14 (9.5%)	
**LABA+LAMA**	1 (0.7%)	33 (22.3%)	
**LABA+ICS**	128 (88.3%)	24 (16.2%)	
**LABA+LAMA+ICS**	7 (4.8%)	64 (43.2%)	
**Presence of family history for airway disease**	71 (49.3%	30 (20.3%)	<0.001
**Mild-moderate exacerbations**			<0.001
**0-1**	127 (87.6%)	101 (68.2%)	
**≥2**	18 (12.4%)	47 (31.8%)	
**Hospitalization requiring exacerbation**			<0.001
**0**	139 (95.9%)	118 (79.7%)	
**≥1**	6 (4.1%)	30 (20.3%)	
**GOLD**			
**A**	-	53 (35.8%)	
**B**	-	44 (29.7%)	
**E**	-	51 (34.4%)	
**Asthma severity**			
**Mild**	37 (25.5%)	-	
**Moderate**	81 (55.9%)	-	
**Difficult to treat**	27 (18.6%)	-	

**Asthma control test score (mean,SD)**	18,2(5.11)	-	
**Very poorly conrolled (5-15)**	41 (28.3%)	-	
**Not-well controlled (16-19)**	34 (23.4%)	-	
**Well-controlled (20-25)**	70 (48.3%)	-	

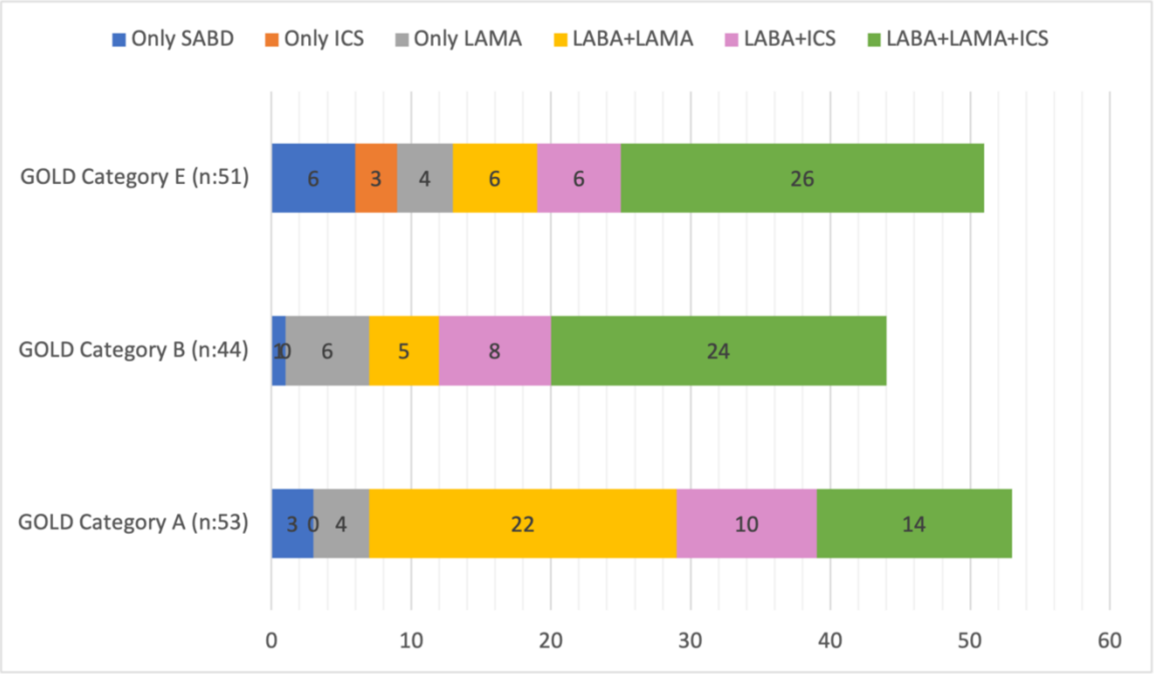

© 2024 Karadogan D. et al.
